# Hemangioblastoma of the Central Nervous System: A Case Series of Patients Surgically Treated at Shohada-e-Tajrish Hospital, Tehran, Iran during 2004-2014

**Published:** 2019

**Authors:** Mahsa AHADI, Hanieh ZHAM, Azadeh RAKHSHAN, Mitra RAFIZADEH, Davood TALEBI BAYAZI, Masoud BAIKPOUR, Afshin MORADI

**Affiliations:** 1 Cancer Research Center, Shohada-e Tajrish Hospital, Shahid Beheshti University of Medical Sciences, Tehran, Iran

**Keywords:** Hemangioblastoma, Surgical pathology, Central nervous system, Location

## Abstract

**Objectives:**

Hemangioblastoma refers to a benign vascular neoplasm that comprises stromal and capillary cells. Based on the classification of nervous system tumors proposed by WHO, hemangioblastomas are classified as Grade I meningeal tumors of uncertain origin. These tumors are found almost exclusively in the central nervous system (CNS) and account for 0.9% to 2.1% of all primary CNS tumors.

**Materials & Methods:**

In this descriptive retrospective study, the archives of pathology reports were reviewed in the Department of Pathology of Shohada-e-Tajrish Hospital, Tehran, Iran and patients with definite diagnosis of hemangioblastoma made through histopathological examinations during 2004-2014 were identified. Age, gender and the location of tumor were extracted from the medical records and entered into SPSS statistical software v.22 for analysis.

**Results:**

Thirty patients including 16 males (53.3%) and 14 females (46.7%) were identified. The mean age of the patients was calculated to be 41.2±13.47 yr, ranging from 19 to 62 yr old. The majority of lesions had been found in the cerebellum of the patients (93.3%); only one had occurred in the cerebrum (3.3%) and another in the fourth ventricle (3.3%).

**Conclusion:**

Cerebellum is the most commonly affected location in patients with CNS hemangioblastomas, and a male preponderance is observed in these cases.

## Introduction

Hemangioblastoma refers to a benign vascular neoplasm that comprises stromal and capillary cells (1). Based on the classification of nervous system tumors proposed by the WHO, hemangioblastomas are classified as Grade I meningeal tumors of uncertain origin (2, 3). These tumors are found almost exclusively in the central nervous system (CNS) and rarely occur in the peripheral nervous system. They can occur sporadically in 60%-75% of cases or in association with von Hippel-Lindau (VHL) disease in 20%-40% of cases (4, 5). Sporadic lesions have a later onset (40-50 yr) compared to VHL-associated tumors (30-40 yr) (6). Hemangioblastomas account for 0.9% to 2.1% of all primary CNS tumors and most commonly affect the cerebellum (63%), followed by spinal cord (32%) (7, 8) and medulla (5%) (9). The tumor has also been rarely reported in other locations including supratentorial compartment (10-12), sella turcica (13), optic nerve (14, 15), ventricular system (16, 17) peripheral nerves (18, 19) or soft tissues (20). Males are 1.5 to 2 times more frequently affected by hemangioblastomas compared to females. 

On neuroimaging studies, the lesions appear as either small contrast-enhancing mural nodules with associated pseudocysts in 30% to 80% of cases, or solid tumors (21). The clinical presentation of hemangioblastoma is caused by the mass effect of the tumor or impairment of cerebrospinal fluid and depends on the anatomical location and growth pattern of the lesion (22-24). Generally, intracranial lesions present with a long history of minor neurological symptoms followed by an abrupt exacerbation. Ataxia, discoordination or increased intracranial pressure can be seen in cerebellar hemangioblastomas and spinal cord lesions can be associated with pain and signs of spinal cord compression. 

Considering the low incidence of hemangioblastoma and the consequent lack of information on this entity particularly in Iran, hereby we present basic demographic characteristics of hemangioblastoma tumors diagnosed in the patients referring to Shohada-e-Tajrish Hospital, Tehran, Iran during 2004-2014. 

## Materials & Methods

In this descriptive retrospective study, the archives of pathology reports were reviewed in the Department of Pathology of Shohada-e-Tajrish Hospital and patients with definite diagnosis of hemangioblastoma made through histopathological examinations during 2004-2014 were identified. Age, gender and the location of tumor were extracted from the medical records and entered into SPSS statistical software v.22 (25) for analysis.

The study was approved by Ethics Committee of the hospital. 

## Results

Thirty patients including 16 males (53.3%) and 14 females (46.7%) were identified. The mean age of the patients was calculated to be 41.2±13.47 yr, ranging from 19 to 62 yr old. The mean age of male subjects was slightly higher than that of females (41.75 vs. 40.57 yr). The majority of lesions had been found in the cerebellum of the patients (93.3%); only one had occurred in the cerebrum (3.3%) and another in the fourth ventricle (3.3%) (Table 1). Overall, 28 lesions found in the cerebellum, 3 (10.0%) were reported in the right hemisphere, 7 (23.3%) in the left hemisphere and the remaining 18 (60.0%) were just reported to be in the cerebellum and their exact location was not recorded. Table 2 presents age- gender and site-wise distribution of the 30 cases included in this study. 

## Discussion

Overall, 30 patients with definite diagnosis of hemangioblastoma were identified in the pathology archives of Shohada-e-Tajrish Hospital. The mean age of the patients was found to be 41.2±13.47 yr, ranging from 19 to 62 yr old, which was quite compatible with the results of previous reports (4, 6, 7, 26, 27). Cerebellum was the most commonly affected location by the tumors accounting for 93.3% of cases. This figure was slightly higher than the reports of previous studies. Overall, 63% of cerebellum involvement was reported in patients with CNS hemangioblastomas (6). The second most common affected site has been reported by multiple studies to be the spinal cord (2, 4, 6, 23, 27) while none of the cases included in our study had tumors of this location. Instead the two sites affected by hemangioblastomas other than cerebellum were found to be cerebrum in one patient and the fourth ventricle in another. A male preponderance was observed in the present case series with a ratio of 1.14. Some of the previous studies had shown equal risk in both genders (28) while other surveys have reported the same male preponderance as found in the present study (4, 6, 7, 29).

Hemangioblastomas are adherent to the pia mater and have a well-defined border. They usually are bright red or red-orange due to the extreme vascularity from the pial vessels (30). The tumor consists of yellow areas of the lipid-laden stromal cells with a spongy and hemorrhagic cut surface. The majority of its vascular components consists comprise small capillaries that may lead to larger vessels. The lesion might also have peritumoral cysts fluid filled and surrounded by Rosenthal fibers and reactive gliosis (6). 

**Table 1:** Demographic characteristics of the patients

Age (yr)		41.2±13.47
Gender	Male	16 (53.3%)
	Female	14 (46.7%)
Site	Cerebellum (Side not specified)	18 (60.0%)
	Cerebellum (Right hemisphere)	3 (10.0%)
	Cerebellum (Left hemisphere)	7 (23.3%)
	Fourth ventricle	1 (3.3%)
	Cerebrum	1 (3.3%)

**Table 2:** Age-gender- and site-wise distribution of hemangioblastoma cases

**Year**	**Age(yr)**	**Gender**	**Site**
2004	52	Female	Cerebellum
2005	59	Female	Cerebellum
	62	Female	Cerebellum
2006	50	Male	Cerebellum (Right hemisphere)
	48	Male	Cerebellum (Left hemisphere)
	62	Female	Cerebellum (Left hemisphere)
2007	23	Male	Cerebellum
	31	Male	Cerebellum
	19	Female	Fourth ventricle
	28	Female	Cerebellum (Right hemisphere)
2008	49	Male	Cerebellum
	24	Female	Cerebellum
	53	Female	Cerebellum
2009	48	Male	Cerebellum
	61	Male	Cerebellum
	23	Male	Cerebellum
	38	Female	Cerebellum (Left hemisphere)
	20	Female	Cerebellum
	39	Female	Cerebellum
	41	Female	Cerebellum (Left hemisphere)
	27	Female	Cerebellum
2010	45	Male	Cerebellum
	34	Male	Cerebellum
2011	42	Male	Cerebellum (Right hemisphere)
	27	Male	Cerebellum (Left hemisphere)
2012	43	Male	Cerebellum (Left hemisphere)
	58	Male	Cerebellum
	32	Male	Cerebrum
2013	44	Female	Cerebellum (Left hemisphere)
2014	54	Male	Cerebellum

As highly vascular tumors, hemangioblastomas can consist of variable proportions of capillary-sized thin walled vessels interweaved with pericytes and inhibin-expressing, round or polygonal, vacuolated stromal cells of uncertain origin (31). These stromal cells are lightly periodic acid-Schiff (PAS) positive and have a lipid-rich, pale eosinophilic cytoplasm. The nuclei of these cells are round or oval and centrally located and might show considerable pleomorphism such as karyomegaly, irregularities of the nuclear membrane and hyperchromasia. Specific organelles or intracellular attachments might lack in these stromal cells but various lipid droplets and glycogen particles might be found in their cytoplasm (32). Weibel-Palade-like bodies and neurosecretory granule-like structures have also been described by some studies. Uncommonly, mitotic figures do not affect the clinical characteristics of the lesion. A second somatic mutation of the VHL allele is present in these stromal cells and so they are considered as the neoplastic element in hemangioblastomas. In the reticular variant of the lesion, vascular components are predominant while the stromal components predominate in the cellular variant (6). 

The treatment of choice for hemangioblastomas is surgical excision and radiotherapy is reserved for non-resectable or recurrent lesions (33). Tumor regrowth might be associated with solid lesions and paucity of stromal cells within the tumor (34). VHL disease has been shown to be accompanied by an increased risk for multifocal hemangioblastomas and extracerebellar lesion (9). 

**In conclusion, **cerebellum is the most commonly affected location in patients with CNS hemangioblastomas, and a male preponderance is observed in these cases. 
